# Combined QTL-Seq and Traditional Linkage Analysis to Identify Candidate Genes for Purple Skin of Radish Fleshy Taproots

**DOI:** 10.3389/fgene.2019.00808

**Published:** 2019-09-20

**Authors:** Tongjin Liu, Jinglei Wang, Chunhui Wu, Youjun Zhang, Xiaohui Zhang, Xiaoman Li, Haiping Wang, Jiangping Song, Xixiang Li

**Affiliations:** ^1^Institute of Vegetables and Flowers, Chinese Academy of Agricultural Sciences, Key Laboratory of Biology and Genetic Improvement of Horticultural Crops, Ministry of Agriculture, Beijing, China; ^2^Institute of Vegetables Research, Zhejiang Academy of Agricultural Sciences, Hangzhou, China

**Keywords:** radish, purple taproot skin, inheritance, QTL-seq, candidate gene

## Abstract

Taproot skin color is a crucial visual and nutritional quality trait of radish, and purple skin is most attractive to consumers. However, the genetic mechanism underlying this character is unknown. Herein, F_2_ segregating populations were constructed to investigate radish genomic regions with purple skin genes. Segregation analysis suggested that pigment presence was controlled by one dominant gene, *Rsps*. A bulk segregant approach coupled to whole-genome sequencing (QTL-seq) and classical linkage mapping narrowed the *Rsps* location to a 238.51-kb region containing 18 genes. A gene in this region, designated *RsMYB1.1* (an *Arabidopsis PAP1* homolog), was a likely candidate gene because semiquantitative RT-PCR and quantitative real-time PCR revealed *RsMYB1.1* expression in only purple-skinned genotypes, sequence variation was found between white- and purple-skinned radishes, and an InDel marker in this gene correctly predicted taproot skin color. Furthermore, four *RsMYB1.1* homologs (*RsMYB1.1-1.4*) were found in “XYB36-2” radish. *RsMYB1.1* and the previously mapped and cloned *RsMYB1.4* (contributing to red skin) were located on different chromosomes and in different subclades of a phylogenetic tree; thus, they are different genes. These findings provide insight into the complex anthocyanin biosynthesis regulation in radish and information for molecular breeding to improve the anthocyanin content and appearance of radish taproots.

## Introduction

Radish (*Raphanus sativus*, 2n = 2x = 18), a member of the Brassicaceae (Cruciferae) family, is an important root vegetable grown and consumed throughout the world. In addition to its root, the leaf, siliques, and seed oil of some cultivars are consumed in some regions. The taproots of radish exhibit significant variation in shape, size, color, and flavor. There are many different taproot skin colors, including purple, red, pink, white, green, and even black and yellow.

The color of agricultural products is an important visual quality trait that directly affects the choice behavior of consumers. For this reason, a number of studies have attempted to identify major genes that control the color of different vegetables by map-based cloning. In cucumber, two genes responsible for fruit color were identified by map-based cloning. A single-nucleotide insertion in *APRR2* was responsible for a white immature fruit color (Liu H. et al., 2016), while a mutation in MYB36 resulted in the formation of a yellow-green peel mutant (Hao et al., 2018). A 4-bp insertion in a putative R3 MYB repressor, *SIMYBATV*, led to anthocyanin accumulation in tomato fruit (Cao et al., 2017). A previous study mapped a purple leaf gene in ornamental kale to a 44.8-kb interval, and a dihydroflavonol reductase-coding gene was identified as a candidate gene (Liu et al., 2017). Through map-based cloning, *BnAPR2* was identified as a candidate gene responsible for the purple leaf trait in *Brassica napus* (Li et al., 2016). More recently, *RsMYB1*, an R2R3-MYB transcription factor, was identified in the red-skinned radish cultivar “Lian Yan No. 1” by map-based cloning (Yi et al., 2018). However, to date, no map-based cloning studies have been performed for the major genes responsible for the purple skin of radish taproots, which is an important visual and nutritional quality character.

In recent years, with an increase in people’s consumption level, red and purple radishes have gained popularity because their enriched glucosinolates and anthocyanins have health benefits (Park et al., 2011). Developing radish cultivars with different skin colors has become an important breeding objective, and illuminating the mechanism of skin color formation will significantly accelerate progress in skin color breeding. Previous studies have indicated that anthocyanin accumulation results in the red/purple skin of radish (Park et al., 2011; Muleke et al., 2017). The anthocyanin biosynthesis pathway is relatively conserved in plants and has been extensively studied (Park et al., 2011; Zhang Y. et al., 2015; Zhang et al., 2015; Chen et al., 2016). The regulation of this process is extremely complex, and some members of the MYB, basic helix–loop–helix (bHLH), WD40, lateral organ boundary domain (LBD), WRKY, and NAC transcription factor families have been reported to be involved in its positive or negative regulation (Johnson et al., 2002; Koes et al., 2005; Matsui et al., 2008; Rubin et al., 2009; Stracke et al., 2010; Zhou et al., 2015). Recently, most structural genes involved in a multi-enzymatic pathway that controls anthocyanin biosynthesis were reported in radish (Park et al., 2011; Chen et al., 2016; Muleke et al., 2017; Sun et al., 2018). However, only two transcription factors (*RsMYB1* and *RsTT8*) that regulate anthocyanin accumulation in radish taproots identified by homology or map-based cloning have been reported (Lim et al., 2016; Lim et al., 2017; Yi et al., 2018).

Some early studies showed that a single locus or multiple loci may control root skin color and that red is dominant over white (Yarnell, 1956; He et al., 1997). He et al. (1997) indicated that red skin was probably controlled by three pairs of independent genes and that some genes controlling skin color likely affected flesh color inheritance (He et al., 1997). In contrast, Yi et al. (2018) reported that red skin was determined by a single dominant gene. These conflicting results most likely occur because the inheritance of radish root skin color is complex, and different inheritance mechanisms may be involved in different materials. The inheritance patterns and chromosomal locations of purple taproot skin genes in radish are still unclear. Therefore, the identification and cloning of major genes responsible for the purple skin of radish deserve further study.

In the present study, the radish inbred line CX16Q-25-2 (purple skin with white flesh) was crossed with the inbred line CX16Q-1-6-2 (white skin and flesh) to construct an F_2_ population, which was used for fine genetic mapping of the genes for purple skin. Furthermore, genes located within the mapping region were analyzed, a candidate gene was identified and named *RsMYB1.1*, and four *RsMYB1.1* homologous genes distributed on three chromosomes of “XYB36-2” radish were identified and named *RsMYB1.1-1.4* according to their chromosome position. These findings will facilitate elucidation of the molecular mechanism for purple skin formation in radish taproots.

## Materials and Methods

### Plant Materials

**Figure 1:**
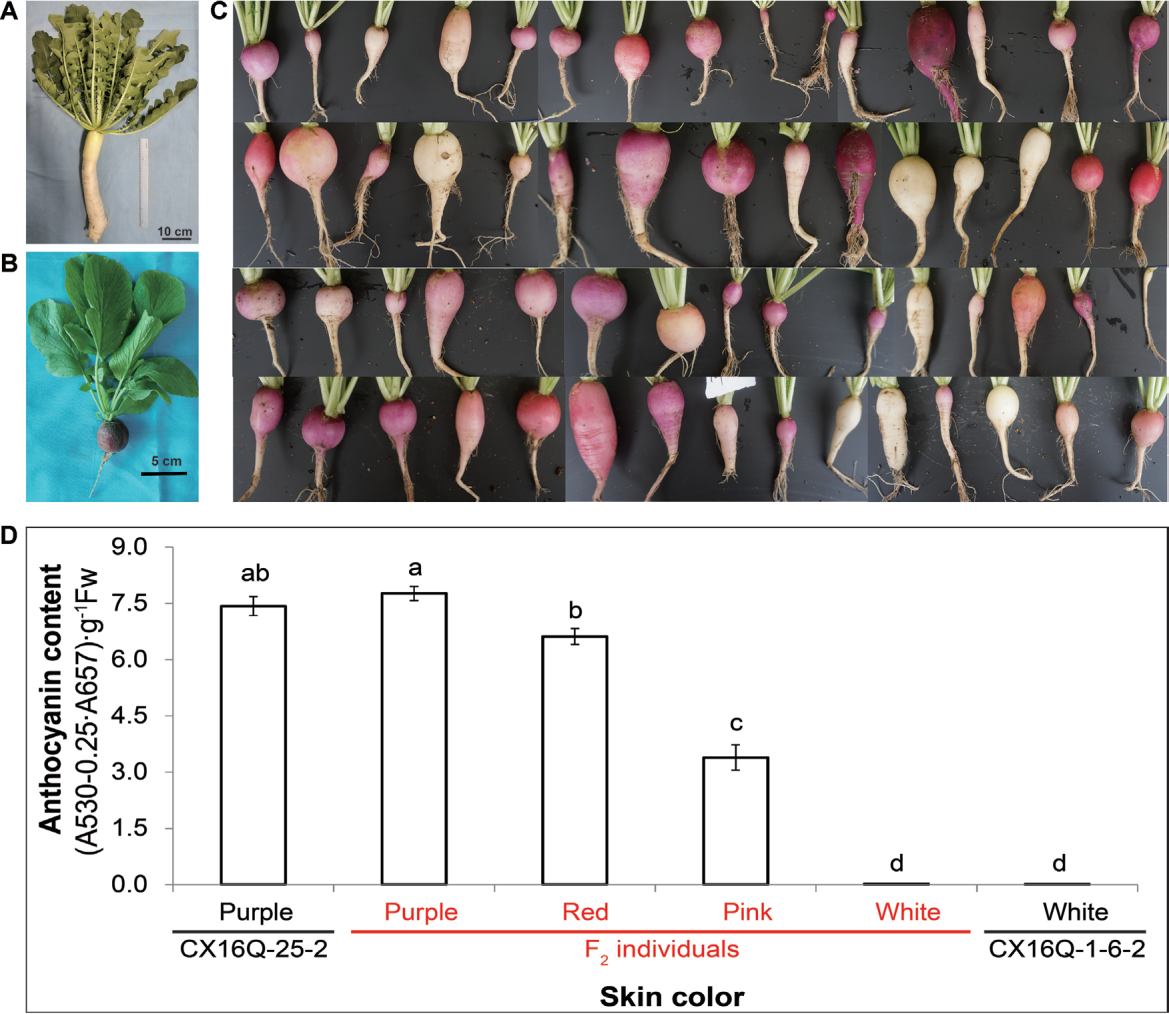
Skin color of the parental lines and their derived F_2_ population. **(A)** CX16Q-1-6-2; **(B)** CX16Q-25-2; **(C)** F_2_ individuals of CX16Q-25-2 × CX16Q-1-6-2 divided into purple skins with varying shades and white skins; **(D)** relative total anthocyanin content of the skin of taproots with different skin colors.

The white-skinned male radish inbred line CX16Q-1-6-2 (**Figure 1A**) was crossed with the purple-skinned female radish inbred line CX16Q-25-2 (**Figure 1B**) to generate the F_1_ generation. F_2_ populations (**Figure 1C**) were generated from self-pollination of a single F_1_ plant and further self-pollinated to generate the F_2:3_ population. All plants were grown at the Institute of Vegetables and Flowers (IVF), Chinese Academy of Agricultural Sciences (CAAS), Beijing, China.

### Phenotyping and DNA Extraction

Skin color was identified visually 40 days after planting. Young leaves of each plant were collected, freeze dried, and ground into a fine powder for DNA extraction. Genomic DNA was extracted from 100 mg of leaf powder of each plant using the CTAB method (Doyle and Doyle, 1987). The quality of the DNA was assessed by 1% agarose gel electrophoresis and a NanoPhotometer (Implen, CA, USA), and the concentrations were measured by a Qubit DNA Assay Kit in a Qubit 2.0 Fluorometer (Life Technologies, CA, USA).

### Total Anthocyanin Content Measurements

Total anthocyanins were extracted from 0.5 g of finely ground taproot skin from CX16Q-25-2, CX16Q-1-6-2, and different F_2_ individuals (purple, red, pink, and white taproot skin) and evaluated by measuring the absorbance of the extract at 530 and 657 nm, as described by Chu et al. (2013). Anthocyanins were quantitated using the following formula: Q = (A_530_ − 0.25 × A_657_) × M^−1^, where Q is the anthocyanin content, A_530_ and A_657_ are the absorbance at the wavelengths 530 and 657 nm, respectively, and M is the weight of the sample. All samples were measured as triplicates in three independent biological replicates. Error bars indicate the standard error (SE) of the average of the anthocyanin content.

### Bulk Segregant Approach Coupled to Whole-Genome Sequencing (QTL-seq) Analysis

For the QTL-seq analysis, a purple-skinned DNA pool (P-pool) and a white-skinned DNA pool (W-pool) were constructed by mixing equal amounts of DNA from 40 purple-skinned and 40 white-skinned F_2_ plants from the 2017 fall experiment, respectively. Four sequencing libraries, namely, P-pool, W-pool, and the two parental inbred lines (CX16Q-25-2 and CX16Q-1-6-2), were prepared for paired-end (150 bp) sequencing with the Illumina HiSeq platform. Clean reads were obtained by trimming adaptors of raw sequence reads (“.fastq” format), removing reads with ambiguous “N” nucleotides exceeding 5% of the read length, and eliminating reads containing a number of bases with a Q value below 5 exceeding 50% of the total length. High-quality clean reads were used for QTL-seq analysis and insertion and deletion (InDel) marker development (Gordon, 2009).

A QTL-seq pipeline (http://genome-e.ibrc.or.jp/home/bioinformatics-team/mutmap, developed by the Iwate Biotechnology Research Center, Japan) was used to identify candidate genomic regions. Briefly, the cleaned reads of CX16Q-25-2 were first aligned to the reference genome “XYB36-2” (Zhang et al., 2015b) using an inbuilt BWA aligner (Li and Durbin, 2009). The reference-guided assembly of the CX16Q-25-2 parent was developed by substituting the bases with high-confidence nucleotides called for the CX16Q-25-2 parent. Then, the reads from the P and W bulks were aligned to the developed assembly, and variants were called for both bulks. The single nucleotide polymorphism (SNP) index for both bulks was calculated at each SNP position as suggested by Hiroki et al. (2013). The ΔSNP index was obtained by subtracting the SNP index of the P-pool from that of the W-pool. The average ΔSNP index located in a certain genomic interval was calculated using a sliding window analysis with a 4-Mb window size and 20-kb increment. Statistical confidence intervals of the ΔSNP index were calculated under the null hypothesis of no quantitative trait locus (QTL) following the description by Takagi et al., and 95% confidence intervals of the ΔSNP index were used to identify genomic regions containing candidate major QTLs (Hiroki et al., 2013).

### QTL Analyses With InDel Markers

QTL-seq analysis identified the major QTL for *Rsps* (purple skin). To fine map the *Rsps* gene, InDel markers were designed within the *Rsps* target region according to the polymorphism information of two resequenced parental inbred lines: CX16Q-25-2 and CX16Q-1-6-2. The short reads from parental inbred lines were aligned to the “XYB36-2” reference genome (Zhang et al., 2015b) with BWA software (Li and Durbin, 2009). InDel calling was performed by SAMtools software (Li and Durbin, 2009). Primer pairs were designed using Primer3 (Triinu and Maido, 2007), and specific primers were selected by alignment with the “XYB36-2” reference genome using electronic PCR (e-PCR) software (Schuler, 1997). Polymorphic markers were screened using the DNA of P-pool, W-pool, and the two parental inbred lines (CX16Q-25-2 and CX16Q-1-6-2) and then applied to the plants in the F2 population from the 2017 fall experiment.

PCR amplifications were performed in a 15-μl volume as described by Zhang et al. (2015a). The primers are listed in **Supplementary Table 1**. The amplified products were separated on an 8% polyacrylamide gel with 1× Tris/borate/EDTA (TBE) buffer at a constant voltage of 180 V for 1 h and then visualized with silver staining. The marker bands of individuals with the CX16Q-25-2, CX16Q-1-6-2, and F_1_ alleles were recorded as “a”, “b”, and “h”, respectively. JoinMap 4.0 was used to perform linkage analysis (Van Ooijen et al., 2002).

### Candidate Gene Identification

To amplify the candidate gene, the DNA sequence was extracted from the radish genome (Zhang et al., 2015b), and a specific PCR primer was designed using Primer3web (Untergasser et al., 2012) (see **Supplementary Table 1**). PCR amplification was performed in a total volume of 50 µl using KOD-Plus-Neo polymerase (Toyobo, Osaka, Japan) according to the manufacturer’s specifications with the two-step cycle procedure (Liu T. et al., 2016). The PCR products were subjected to Sanger sequencing using an ABI 3730 instrument (Applied Biosystems, CA, USA).

### RNA Extraction, Semiquantitative RT-PCR, and Quantitative Real-Time PCR (qPCR) of the Candidate Gene

Total RNA was extracted from the taproot skin of CX16Q-25-2, CX16Q-1-6-2, and different F_2_ individuals (purple, red, pink, and whitetaproot skin), treated with DNase and then reverse transcribed into cDNA as previously described (Liu T. et al., 2016). The specific primer was designed using Primer3web (Untergasser et al., 2012) and is shown in **Supplementary Table 1**. Semiquantitative RT-PCR for the candidate gene was carried out between CX16Q-25-2 and CX16Q-1-6-2 in a total volume of 50 µl using KOD-Plus-Neo polymerase with a T100 thermal cycler (Bio-Rad, Hercules, CA, USA). The following cycling parameters were used: 2 min of predenaturation at 94°C, followed by 35 cycles of denaturation (98°C, 10 s), annealing (63°C, 30 s), and extension (68°C, 30 s). Ten microliters of PCR product was then separated on a 2% agarose gel stained with GoldView. To investigate the pigment-controlling role of the candidate gene, qPCR analysis was performed in the taproot skin of parental lines and different F_2_ individuals (purple, red, pink, and white). The cDNA was diluted fivefold as templates for qPCR using SuperReal PreMix Plus (TIANGEN BIOTECH CO., LTD, Beijing, China). The reaction volume was 20 μl, including 0.6 μl of forward and reverse primers at 10 mM each, 10 μl of 2 × SuperReal PreMix Plus, 2.0 μl of the cDNA sample, 2.0 μl of 50 × ROX Reference Dye, and 4.8 μl of ddH_2_O. The thermal cycling profile was 95°C for 15 min; 40 cycles of 95°C for 10 s, 60°C for 30 s; then 95°C for 15 s, 60°C for 1 min, and ramping to 95°C for 15 s. Three independent biological and technical replicates were performed. Data were analyzed using StepOne™ Software v.2.0 (Applied Biosystems). Radish glyceraldehyde 3-phosphate dehydrogenase (*RsGADPH*) transcripts were used as a control for semiquantitative RT-PCR and an internal reference for qPCR (Liu et al., 2019).

### Search for Homologs of the Candidate Gene

The predicted protein sequence of the candidate gene was used to search for corresponding homologs by BLASTP with previously published radish genome data (Zhang et al., 2015). Related protein sequences of other species were obtained from the NCBI database (http://www.ncbi.nlm.nih.gov/). DNAMAN8 software (Lynnon, Quebec, Canada) was used to align sequences with the default parameters, and MEGA5 software was used to generate a phylogenetic tree according to the predicted protein sequences (Tamura et al., 2011).

## Results

### Inheritance of the Purple Skin of Radish Taproots

To determine the inheritance of purple skin in radish, purple-skinned (CX16Q-25-2) and white-skinned (CX16Q-1-6-2) radish inbred lines were used to generate F_1_ and F_2_ populations (**Figure 1**). The fleshy taproot skin color of all F_1_ individuals was purple (**Table 1**). The 557 F_2_ individuals exhibited complex skin color segregation including white, pink, red, and purple of different shades. The total anthocyanin content of the parental lines and different F2 radish roots are significantly different (**Figure 1D**). This result indicated that the genetics of purple taproot skin were complex, with multiple factors involved. However, the segregation was consistent with a 3:1 Mendelian segregation ratio for pigmented (pink, red, and purple with different shades) and white roots, which indicated that pigment presence was controlled by a single dominant gene, designated *Rsps* (purple skin). The present study was mainly focused on the identification of this dominant gene.

**Table 1:** Segregation of taproot skin color in the F_1_ and F_2_ populations.

Population	Plants tested	Pigmented: white	Mendelian expectation	χ2 value^a^	*p*
CX16Q-25-2 ×CX16Q-1-6-2							
F_1_	all	Pigmented	1:0		
F_2_	557	419:138	3:1	0.33	0.57

CX16Q-25-2 and CX16Q-1-6-2 are radish inbred lines with purple and white skin, respectively.

a *χ*2 (0.05, 1) = 3.84

### Preliminary Mapping of *Rsps* by QTL-seq

**Figure 2:**
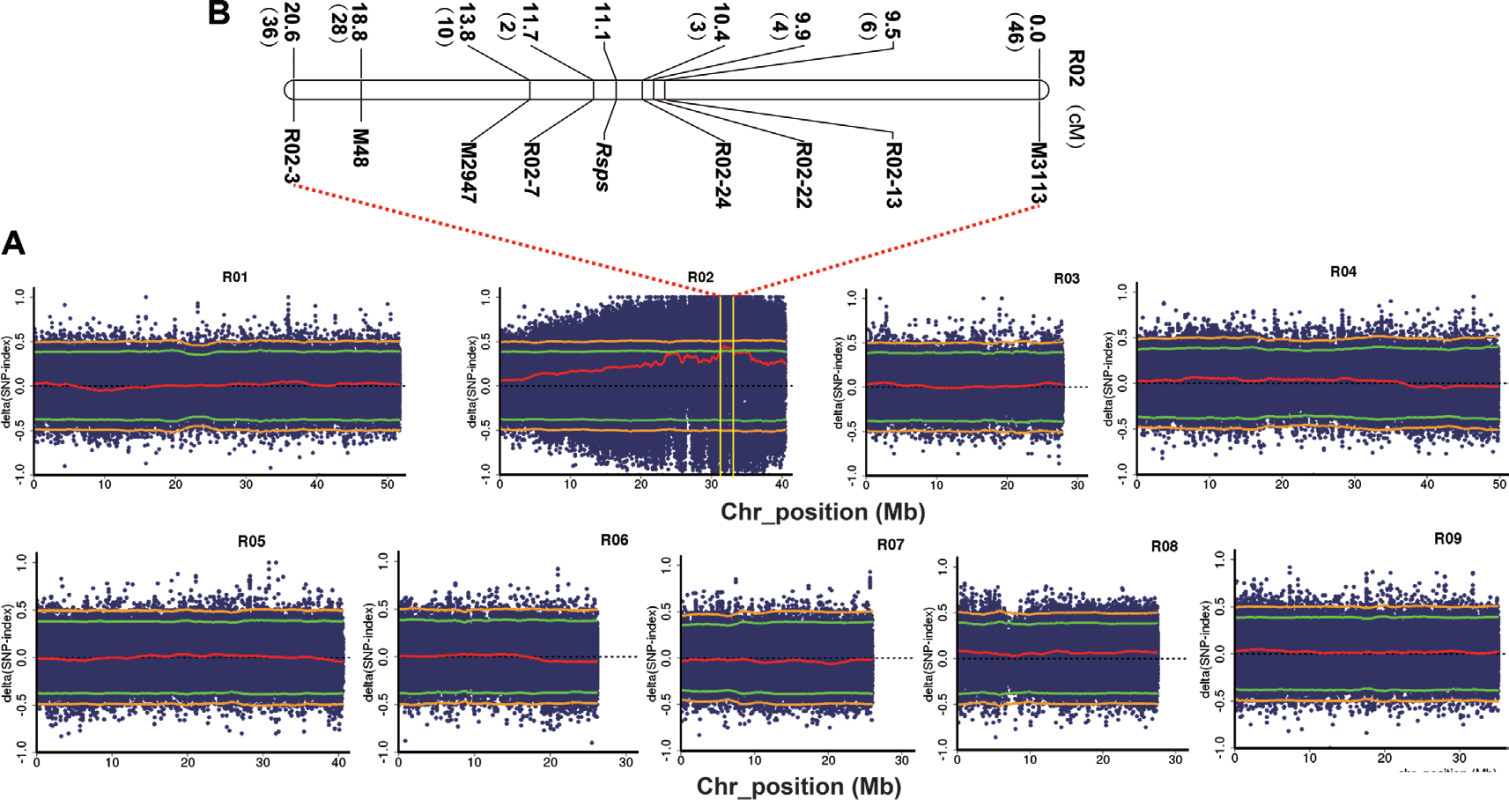
Combined QTL-seq and traditional linkage analysis to map a purple skin locus (*Rsps*) in radish. **(A)** Δ(SNP index) plot of the purple and white bulks with confidence intervals under the null hypothesis of no QTLs (green, *p* < 0.05; orange, *p* < 0.01); **(B)** genetic linkage analysis with InDel markers confirming the position of *Rsps*. The numbers in brackets refer to the number of recombinants of each marker.

The two parental lines (CX16Q-25-2 and CX16Q-1-6-2) and two extreme pools (purple-skinned pool (P-pool) and white-skinned pool (W-pool)) from the F_2_ population were paired-end (150 bp) sequenced with an Illumina HiSeq platform. In total, 5.6, 5.9, 23.4, and 30.0 Gb of clean reads from CX16Q-25-2 (11.2× depth coverage), CX16Q-1-6-2 (11.8× depth coverage), P-pool (46.8× depth coverage), and W-pool (60× depth coverage) were generated, respectively. These short reads were aligned to the “XYB36-2” reference genome, and 1,330,575, 1,130,541, 2,406,284, and 2,452,285 SNPs were identified between CX16Q-25-2 and “XYB36-2,” CX16Q-1-6-2, and “XYB36-2,” P-pool and “XYB36-2,” W-pool and “XYB36-2,” respectively. Subsequent QTL-seq analysis identified only one genomic region from 31.45 to 33.00 Mb on “XYB36-2” radish chromosome 2 that had a Δ(SNP index) value that was significantly different from 0 at the 95% confidence level (**Figure 2A**). These results indicated that there was a major QTL controlling purple skin in the 31.45- to 33.00-Mb region on chromosome 2 in radish (**Figure 2A**), which was designated *Rsps*.

### QTL Analyses With InDel Markers

To verify the accuracy of QTL-seq in the detection of *Rsps* and narrow down its location, traditional QTL analysis was carried out with 352 F_2_ plants. Three polymorphic InDel primer pairs were identified from markers developed in our previous studies (**Supplementary Table 1**). In addition, a total of 674 pairs of InDel primers were developed in the preliminary mapped regions with potential variation of >4 bp (**Supplementary Table 2**). Of the pairs, 52 were synthesized and used to screen polymorphic markers between CX16Q-25-2 and CX16Q-1-6-2 and between P-pool and W-pool, and five markers were determined to be useful (**Supplementary Table 1**). Then, these eight polymorphic markers were applied to the F_2_ populations for QTL mapping. The *Rsps* locus was localized to a 1.3-cM interval between R02-7 and R02-24, flanking the gene at genetic distances of 0.6 cM and 0.7 cM, and this region matched that from QTL-seq (**Figure 2B**). Based on the marker locations of R02-7 and R02-24 in the reference genome of “XYB36-2,” the 238.51-kb interval (31584238–31822749 bp) on chromosome 2 harbors the candidate gene for *Rsps*.

### Candidate Gene Prediction for Purple Taproot Skin in Radish

There were 18 genes within the 238.51-kb candidate region in the “XYB36-2” reference genome (**Table 2**) (http://brassicadb.org/brad/datasets/pub/Genomes/Raphanus_sativus/) (Zhang et al., 2015b). Based on the functional annotations, *Rsa10008423* was predicted to be a member of the MYB family of transcription factors. Blast alignment results showed that the coding sequence (CDS) identity of *Rsa10008423* with the *Arabidopsis* PRODUCTION OF ANTHOCYANIN PIGMENT1 (*AtPAP1*, *AT1G56650*) gene was up to 82.40% (**Supplementary Figure1A**), and the predicted encoded proteins had 77.51% sequence identity (**Supplementary Figure 1B**). *AtPAP1* was reported to be involved in regulating the expression of anthocyanin biosynthetic genes (Borevitz et al., 2000). *Rsa10008423* is a homologous gene of *AtPAP1* in radish; therefore, *Rsa10008423* was designated as *RsMYB1.1*.

**Table 2:** Annotation of radish genes in the candidate region.

Radish genes^a^	Gene position on R02^b^	AT ID^c^	E-value	AT GO annotation^d^
Rsa10008436	31582579-31584948 bp	AT1G66170	0.0	PHD domain-containing protein required for male meiosis
Rsa10008435	31595569-31596809 bp	AT1G66190	1e-52	Hypothetical protein
Rsa10008434	31601403-31606044 bp	AT1G07440	e-115	NAD(P)-binding Rossmann-fold superfamily protein
Rsa10008433	31618713-31620726 bp	AT1G66200	0.0	Cytosolic glutamate synthetase
Rsa10008432	31627059-31652390 bp	AT5G48720	1e-06	XRI1 (X-ray-induced 1)
Rsa10008431	31653659-31654085 bp	AT2G41520	7e-30	One of the 36 carboxylate clamp-tetratricopeptide repeat proteins
Rsa10008430	31678702-31680338 bp	AT3G46920	0.22	Kinase superfamily with an octicosapeptide/Phox/Bem1p domain-containing protein
Rsa10008429	31686259-31688854 bp	AT1G66250	0.0	O-glycosyl hydrolase family 17 protein
Rsa10008428	31689231-31689558 bp	AT2G01918	2e-24	Encodes a protein homologous to each PQL protein
Rsa10008427	31690647-31693371 bp	AT1G66330	e-137	Senescence-associated family protein
Rsa10008426	31696303-31698148 bp	AT1G66345	0.0	pentatricopeptide repeat protein involved in splicing of the nad4 intron, which affects biogenesis of the respiratory complex I
Rsa10008425	31714251-31715573 bp	AT1G17690	3e-31	U3 small nucleolar RNA-associated protein
Rsa10008424	31716933-31718411 bp	AT1G66350	0.0	Negative regulator of gibberellic acid responses, member of the GRAS family of transcription factors
Rsa10008423	31734491-31736148 bp	AT1G56650	1e-103	MYB domain-containing transcription factor involved in anthocyanin metabolism and radical scavenging
Rsa10008422	31737844-31741218 bp	AT1G65320	2e-29	Cystathionine beta-synthase family protein
Rsa10008421	31777128-31778529 bp	AT1G66470	1e-92	ROOT HAIR DEFECTIVE6
Rsa10008420	31805026-31806054 bp	AT2G29880	2e-65	Myb/SANT-like DNA-binding domain protein
Rsa10008419	31815194-31816694 bp	AT3G44840	e-125	S-adenosyl-L-methionine-dependent methyltransferase superfamily protein

a Radish genes in the candidate region of the reference genome “XYB36-2”.

b The physical position of genes in the candidate region of the reference genome “XYB36-2”.

c The best hits for the genes in the candidate region compared to Arabidopsis thaliana (AT).

d Gene Ontology(GO) annotation of the radish genes in the candidate region matched to AT best-hit genes obtained from TAIR (https://www.arabidopsis.org/).

### Analysis of the Candidate Gene

**Figure 3:**
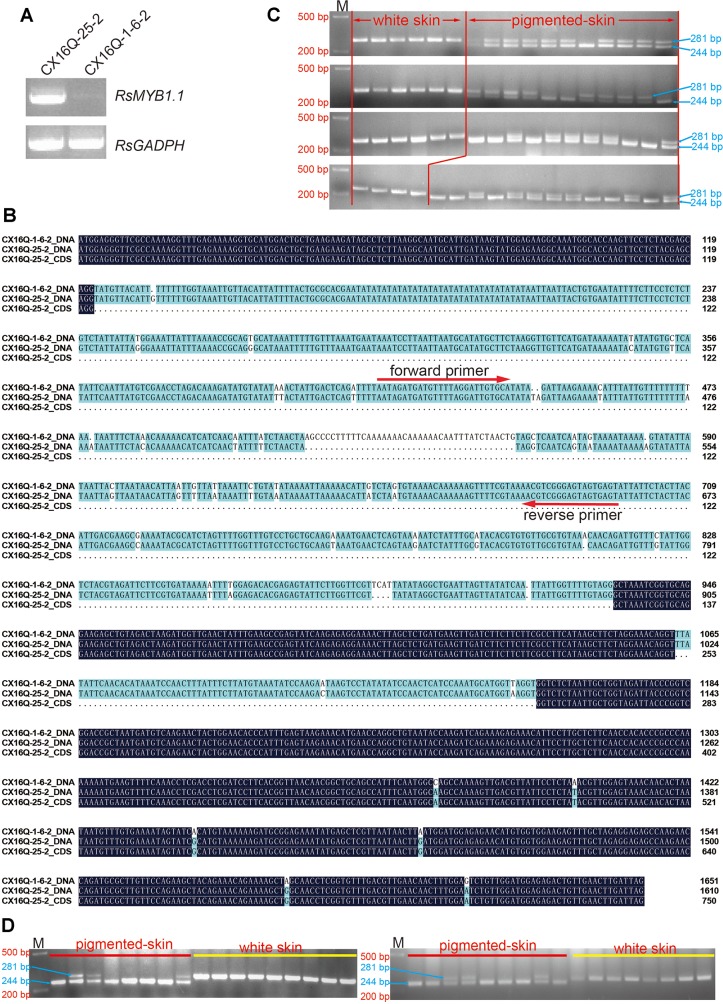
Analysis of candidate genes. **(A)** Expression patterns of *RsMYB1.1* with semiquantitative RT-PCR in the two parental lines; **(B)** alignment of *RsMYB1.1* sequences of white and purple radishes; the positions of the forward and reverse primer pairs designed according to the parental sequence variation used to genotype the F_2_ individuals are shown with arrows; **(C)** genotypes of F_2_ individuals for PCR markers; **(D)** genotypes of F_2:3_ individuals for PCR markers. M, size marker.

We investigated the expression patterns of *RsMYB1.1* with semiquantitative RT-PCR in two parental lines to analyze whether the expression level of *RsMYB1.1* may contribute to the variance in taproot skin color (**Figure 3**). The *RsMYB1.1* transcript was detected only in the taproot skin of purple-skinned radish (**Figure 3A**).

The DNA sequences of the two parental lines and the CDS of *RsMYB1.1* in CX16Q-25-2 were cloned, sequenced, and analyzed to investigate their sequence differences (**Figure 3B**). The alignment of *RsMYB1.1* DNA and CDSs revealed multiple mutations (**Figure 3B**). The DNA sequence is 1,610-bp long, with a 750-bp predicted open reading frame encoding a polypeptide of 249 amino acids. *RsMYB1.1* consists of three exons and two introns, and the first intron (which consists of 769 nucleotides) is significantly longer (8.5 times) than the second intron and represents 47.8% of the total length of the gene. The first and second exons are highly conserved between the two parental lines, while six SNP mutations were found in the third exon. In addition, two SNP mutations were found in the second intron. More notably, the first intron exhibited more SNP and InDel mutations between the two parental lines. Accordingly, an InDel marker was designed in this region, as shown in **Figure 3B**. Forty-six pigmented-skinned (pink, red, and purple with different shades) and 22 white-skinned radishes were selected from the F_2_ population to determine the consistency between these marker genotypes and the taproot skin color phenotypes. Furthermore, the present InDel marker was validated in 17 randomly selected pigmented- and white-skinned radishes from the F_2:3_ population. The results showed that all taproots were predicted correctly (**Figures 3C, D**).

**Figure 4:**
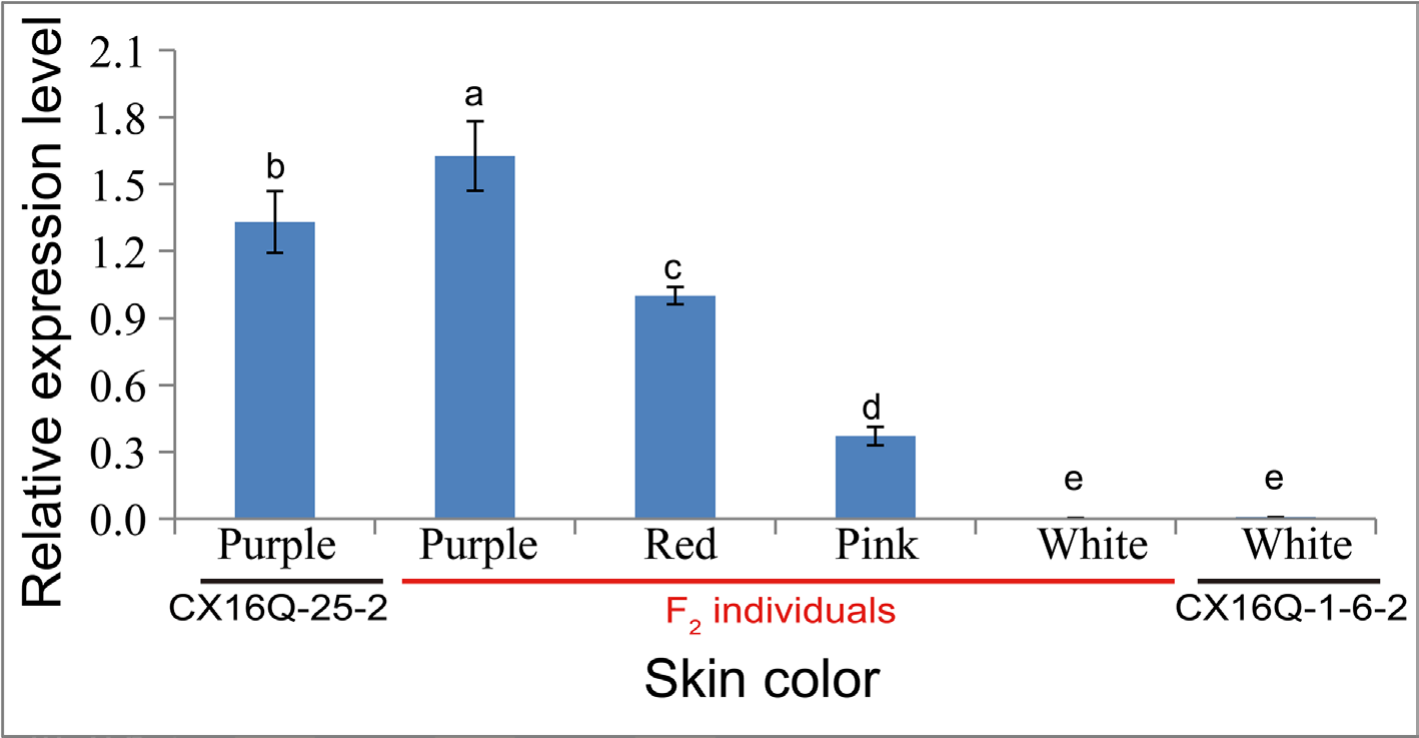
Relative expression of *RsMYB1.1* in the skin of taproots with different skin colors detected by quantitative real-time PCR analysis. (a-e) indicate that the expression values were significantly different.

The expression levels of *RsMYB1.1* in the taproot skin of both parents and F_2_ individuals with purple, red, pink, and white skin were measured by qPCR. *RsMYB1.1* had significantly higher expression levels in pigmented-skinned individuals than in white taproot skin (**Figure 4**). Among pigmented-skinned individuals, purple-skinned plants had the significantly highest expression levels of *RsMYB1.1*, red-skinned plants had the second highest levels, and pink-skinned plants had the significantly lowest expression. The expression trend of *RsMYB1.1* in the taproot skin of parents and F_2_ individuals correlated with their anthocyanin accumulation (**Figures 1** and **4**). These results suggest that *RsMYB1.1* may be a candidate gene controlling purple skin in CX16Q-25-2 radish.

### *MYB1* Genes Were Expanded in the Radish Genome

**Figure 5:**
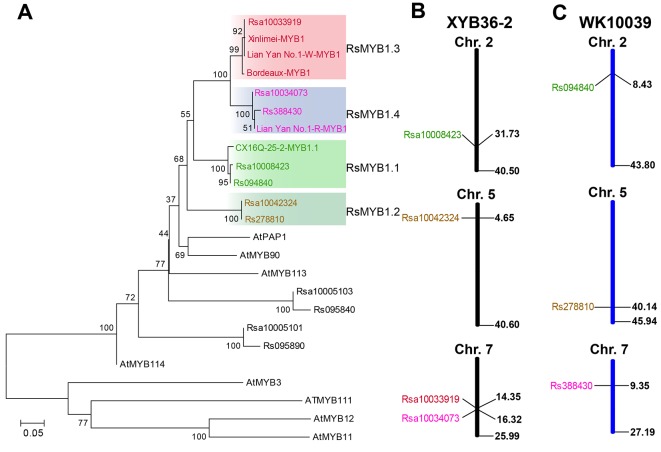
Phylogenetic tree of RsMYB1.1 homologous genes in radish and anthocyanin R2R3-MYB regulators in *Arabidopsis* based on their amino acid sequences. **(A)** The chromosome locations of these genes in the “XYB36-2” **(B)** and “WK10039” **(C)** radish genomes. GenBank accession numbers are as follows: AtMYB11 (NP_191820), AtMYB12 (NP_182268.1), AtMYB75 (NP_176057.1), AtMYB90 (NM_105310), AtMYB111 (NP_199744), AtMYB114 (NP_176812), and Bordeaux-MYB1 (AKM95888.1).

Four and three genes were identified by homology searches in “XYB36-2” (Zhang et al., 2015b) and “WK10039” (Jeong et al., 2016) protein datasets, respectively, with the deduced protein sequence of *RsMYB1.1* from CX16Q-25-2 as a reference. All of these homologous genes were best matched to *Arabidopsis* PRODUCTION OF ANTHOCYANIN PIGMENT1 (*AtPAP1*, *AT1G56650*) in The Arabidopsis Information Resource (TAIR) database (https://www.arabidopsis.org/). In a phylogenetic tree of anthocyanin-regulated R2R3-MYB proteins from *Arabidopsis* and radish, four “XYB36-2” and three “WK10039” homologous genes and the published radish gene MYB1 all fell in the AtPAP1 subgroup (**Figure 5A**). Sequence alignments showed 96.79%, 99.60%, and 100% identity at the amino acid level between Rsa10034073 and Rs388430, Rsa10008423 and Rs094840, and Rsa10042324 and Rs278810, respectively (**Supplementary Figure 2**), indicating that these three gene pairs were most likely the same gene in different radish cultivars. In addition, Rsa10033919 was identified in the “XYB36-2” radish genome but not in the “WK10039” genome (**Figures 5B, C**). This gene falls within the same subclade as the previously reported MYB1 genes from the “Xinlimei” (green skin with red flesh) and “Bordeaux” (red skin with red flesh) radish cultivars and red-skinned “Lian Yan No. 1” radish progeny, indicating that Rsa10033919 is a homolog of RsMYB1. Therefore, according to their chromosome locations in the “XYB36-2” genome, *Rsa10008423* (*Rs094840*), *Rsa10042324* (*Rs278810*), *Rsa10033919*, and *Rsa10034073* (*Rs388430*) were designated *RsMYB1.1*, *RsMYB1.2*, *RsMYB1.3*, and *RsMYB1.4*, respectively.

## Discussion

Fleshy taproot skin color is an important visual and nutritional quality trait of radish that influences radish production and consumption. The identification and cloning of major genes responsible for the skin color of radish will promote effectiveness in the breeding process. To date, possible inheritance patterns of radish taproot skin color have been reported by numerous studies (Yarnell, 1956; He et al., 1997). In different hybrid combinations, green root skin might be controlled by one or two pairs of independent genes or two pairs of linked genes (He et al., 1997). Previous research also indicated that a single locus or multiple loci may control red root skin, and red is dominant over white (Yarnell, 1956; He et al., 1997; Yi et al., 2018). In the present study, a cross between purple (CX16Q-25-2) and white (CX16Q-1-6-2) inbred lines produced F_2_ plants with complex skin color segregation, which was consistent with a previous report that the genetics of purple coloration are complex and involve multiple factors (He et al., 1997). However, because the segregation of pigmented and white roots was consistent with a 3:1 Mendelian segregation ratio, pigment presence was controlled by a single dominant gene. This hypothesis was also verified by subsequent QTL-seq analysis results with two extreme pools (purple- and white-skinned pools) in which only one QTL region was detected. The results are consistent with the finding of a recent study that a single genetic factor distinguishes between red- and white-skinned progenies derived from the Chinese commercial F_1_ cultivar “Lian Yan No. 1” (red skin with white flesh) (Yi et al., 2018). Therefore, our present study mainly focused on the identification of candidate genes located in this QTL region.

We used QTL-seq combined with classical methods to rapidly identify a major QTL, *Rsps*, which was localized to a 238.51-kb physical interval on chromosome 2 (**Figure 3**). Only 18 genes are located in this interval, based on the “XYB36-2” reference genome (**Table 2**). The present study indicated that combined QTL-seq, traditional linkage analysis, and high-quality whole-genome re-sequencing can significantly promote progress in gene mapping and subsequent cloning.

The taproot skins of some radish cultivars are purple and red because of the accumulation of a large concentration of anthocyanins (Park et al., 2011; Yi et al., 2018). The biosynthesis of anthocyanins is regulated by some transcription factors, especially members of the MYB gene family. Among the 18 genes located in the 238.51-kb candidate region of *Rsps* (**Table 2**), the expression pattern (**Figures 3A** and **4**), gene structure (**Figure 3B**), segregation analysis (**Figures 3C, D**), and phylogenetic analysis (**Figure 5A**) of *RsaMYB1.1* suggested that it could be a candidate gene for *Rsps*. RsaMYB1.1 shared 77.51% amino acid sequence identity with *Arabidopsis* AtPAP1 and was expressed in purple-skinned radish plants but not in white-skinned ones. There are multiple mutations in *RsaMYB1.1* between the two parental lines, and the marker designed according to a variable locus separated the purple-skinned and white-skinned radishes correctly (**Figures 3C, D**). Therefore, it is reasonable to postulate that *RsaMYB1.1* is the candidate gene for purple taproot skin in radish. However, further functional research is needed to validate the present findings. The MBW complex composed of R2R3-MYB, basic helix–loop–helix, and WD40 is highly conserved in plants involved in the regulation of anthocyanin biosynthesis (Koes et al., 2005). Among MBW complex components, R2R3-MYB plays a more important role because it is thought to directly bind to the promoter sequences of anthocyanin biosynthesis genes and activate their expression (Hichri et al., 2011). Numerous studies have reported that *MYB* homologs are involved in regulating the biosynthesis of anthocyanin in many plant species, including radish (Koes et al., 2005). A subgroup of *Arabidopsis* R2R3-MYBs including *AtPAP1* (*AtMYB75*), *AtPAP2* (*AtMYB90*), *AtMYB114*, and *AtMYB113* demonstrates a high degree of amino acid conservation and is involved in anthocyanin regulation (Allan et al., 2008). *MdMYBA*, *MdMYB1*, and *MdMYB10* are reportedly responsible for apple anthocyanin accumulation (Takos et al., 2006; Ban et al., 2007; Espley et al., 2007). *BoMYB2* is involved in controlling anthocyanin biosynthesis in purple cauliflower (Chiu and Li, 2012). *SmMYB1* is reported to participate in the regulation of anthocyanin biosynthesis in the peel of eggplant (Zhang et al., 2014). In radish, *RsMYB1* is reported to function in the activation of anthocyanin biosynthesis (Lim et al., 2016; Yi et al., 2018). Therefore, it is not surprising that an *AtPAP1* homologous gene was identified as the candidate gene responsible for the purple skin of CX16Q-25-2 radish.

A previous study suggested that the taproot color of radish was determined by the ratio of pelargonidin and cyanidin, which are responsible for the red and purple coloration, respectively, and the *H* locus was proposed to be involved in the biosynthesis of pigments from pelargonidin to cyanidin (Yi et al., 2018). However, our present research did not detect a QTL region responsible for this trait. The most likely reason for this lack of detection was that the purple or red skin trait in the present F_2_ population was easily affected by environmental conditions. The F_2_ population generated by red- and purple-skinned radish inbred lines may be ideal for mapping the *H* locus.

Four *RsMYB1.1* homologs were identified in the “XYB36-2” radish genome and named *RsMYB1.1*, *RsMYB1.2*, *RsMYB1.3*, and *RsMYB1.4* (**Figure 5B**). *RsMYB1.1* was first reported in the present study and may function in the regulation of anthocyanin biosynthesis in radish taproot skin. The function of *RsMYB1.2* is still unknown and requires further research. In the phylogenetic analysis, the RsMYB1.3 subclade included the previously reported *RsMYB1* gene from the “Bordeaux” homozygous F_3_ progeny (Lim et al., 2016) and “Xinlimei” (Liu et al., 2019), both of which have red flesh. These results indicate that RsMYB1.3 plays an important role in the regulation of anthocyanin biosynthesis in radish taproot flesh. More recently, another *RsMYB1*gene was identified in the red-skinned radish cultivar “Lianyan No. 1” by map-based cloning (Yi et al., 2018). This gene falls in the RsMYB1.4 subclade with Rsa10034073 from “XYB36-2” and Rs388430 from “WK10039”. *RsMYB1.4* and *RsMYB1.1* are located on different chromosomes and fall into different subclades, indicating that they are two different genes, but they might have the same function in the regulation of anthocyanin biosynthesis in radish taproot skin. The identification of different genes previously in “Lianyan No. 1” and in CX16Q-25-2 in the present study may be attributable to the fact that different cultivars were used in mapping candidate genes. Further research is needed to confirm the role of these genes, which would enrich the research on anthocyanin biosynthesis regulation in radish.

## Data Availability

Sequences data generated in this study are available on the Sequence Read Archive http://www.ncbi.nlm.nih.gov/Traces/sra/, at accession PRJNA563272.

## Author Contributions

XXL conceived and supervised the study. TL, JW, and CW analyzed the data and drafted the manuscript. TL, XXL, HW, and JS performed the experiments. JW, TL, and XML helped with mapping population construction and sample collection. XXL, YZ, XZ, and JW revised the manuscript. All authors have read and approved the manuscript.

## Funding

This work was supported by a grant from the National Key Research and Development Program of China (2016YFD0100204-2), the National Natural Science Foundation of China (31801858), the China Postdoctoral Science Foundation Funded Project (2017M620971), and the Science and Technology Innovation Program of the Chinese Academy of Agricultural Sciences (CAAS-XTCX2016016-4-4, CAAS-XTCX2016001-5-2, and CAAS-XTCX2016017).

## Conflict of Interest Statement

The authors declare that the research was conducted in the absence of any commercial or financial relationships that could be construed as a potential conflict of interest.

## Supplementary Material

The Supplementary Material for this article can be found online at: https://www.frontiersin.org/articles/10.3389/fgene.2019.00808/full#supplementary-material

Supplementary Table 1 The primers used in the present study.

Supplementary Table 2 InDel primers developed in the preliminary mapping regions with potential variation of >4 bp.

Supplementary Figure 1 Alignment of radish Rsa10008423 with the Arabidopsis PAP1 (MYB75, AT1G56650) gene coding sequence (a) and their deduced amino acid sequences (b).

Supplementary Figure 2 Alignment of radish Rsa10034073 and Rs388430 (a), Rsa10008423 and Rs094840 (b), and Rsa10042324 and Rs278810 (c) based on their deduced amino acid sequences.
