# Supplementary material for: Combined QTL-Seq and Traditional Linkage Analysis to Identify Candidate Genes for Purple Skin of Radish Fleshy Taproots
Source: Front Genet. 2019 Sep 20;10:808. doi: 10.3389/fgene.2019.00808 (PMC6764292; doi:10.3389/fgene.2019.00808)
Supplement: Supplementary Table 1 — The primers used in the present study. [file Table_1.docx]

| Primer name | Forward Primer (5’-3’) | Reverse Primer (5’-3’) | Description |
| --- | --- | --- | --- |
| M3113 | ACAATCGTATGGATTGCGAA | TTCCTAGCATCAAACGGGAG | Linkage analysis |
| M2947 | TACGACATGCACGGGAATAA | CAAGCAAGCACGTGTCAACT |  |
| M48 | TGTTGGTTCAGTGCCATCAG | AACTGACCATTAGGGAGGGG |  |
| R02-3 | GTCTTCGAAGGGTTTGCCTA | ACCACAAACTCCACCAAACC |  |
| R02-7 | AGCAACCGAAGATCCAAAGA | GGGATCGAATTTGCACTAGC |  |
| R02-13 | AGCCAAAGCTTGAGACCAAA | TCTCGTTGCACTTTCATTGC |  |
| R02-22 | TCACTAGGGATGGGCTTTTG | CCCGCAAATATCCGAATAGA |  |
| R02-24 | GCACCTGGATGCAGAATCTAT | TTTATATCAATAGTGAAGCGTGTTTT |  |
| Rsa10008423F1/R3 | CCCTCTTGTAATCATATCTGCCACC | CAACCATCTTAGTCTACAGCTCTTCC | *RsMYB1.1* full length clone |
| Rsa10008423F4/R4 | GTAAAACGTCGGGAGTAGTGAGT | AACTCATTGCGCTCGACCTTTT |  |
| Rsa10008423cDNA | ATGGAGGGTTCGCCAAAAGGTT | CTAATCAAGTTCAACAGTCTCTCCATCC | *RsMYB1.1* coding sequence clone and semiquantitative RT-PCR |
| GAPDH | GTCAACGATCCTTTCATCACCACTG | ATAGCCTTCTTGATCTCGTCGTAGG | control for semiquantitative RT-PCR |
| Rsa10008423q | TTCTAGGAAACAGGTGGTCTCT | TGGTATTACAGCCTGGTTCATG | quantitative real-time PCR of *RsMYB1.1* |
| Rsa10010156q | ACCACTAACTGCCTTGCTCC | AGCTCTTCCACCTCTCCAGT | internal reference for quantitative real-time PCR |
| MYB1.1-InDel | AATAGATGATGTTTTAGGATTGTGCA | ACTCACTACTCCCGACGTTT | Genotype analysis of F_2_ and F_2:3_ |

Supplementary Table 1. The Primers used in the present study
