# Supplementary figures and images for: Combined QTL-Seq and Traditional Linkage Analysis to Identify Candidate Genes for Purple Skin of Radish Fleshy Taproots
Source: Front Genet. 2019 Sep 20;10:808. doi: 10.3389/fgene.2019.00808 (PMC6764292; doi:10.3389/fgene.2019.00808)

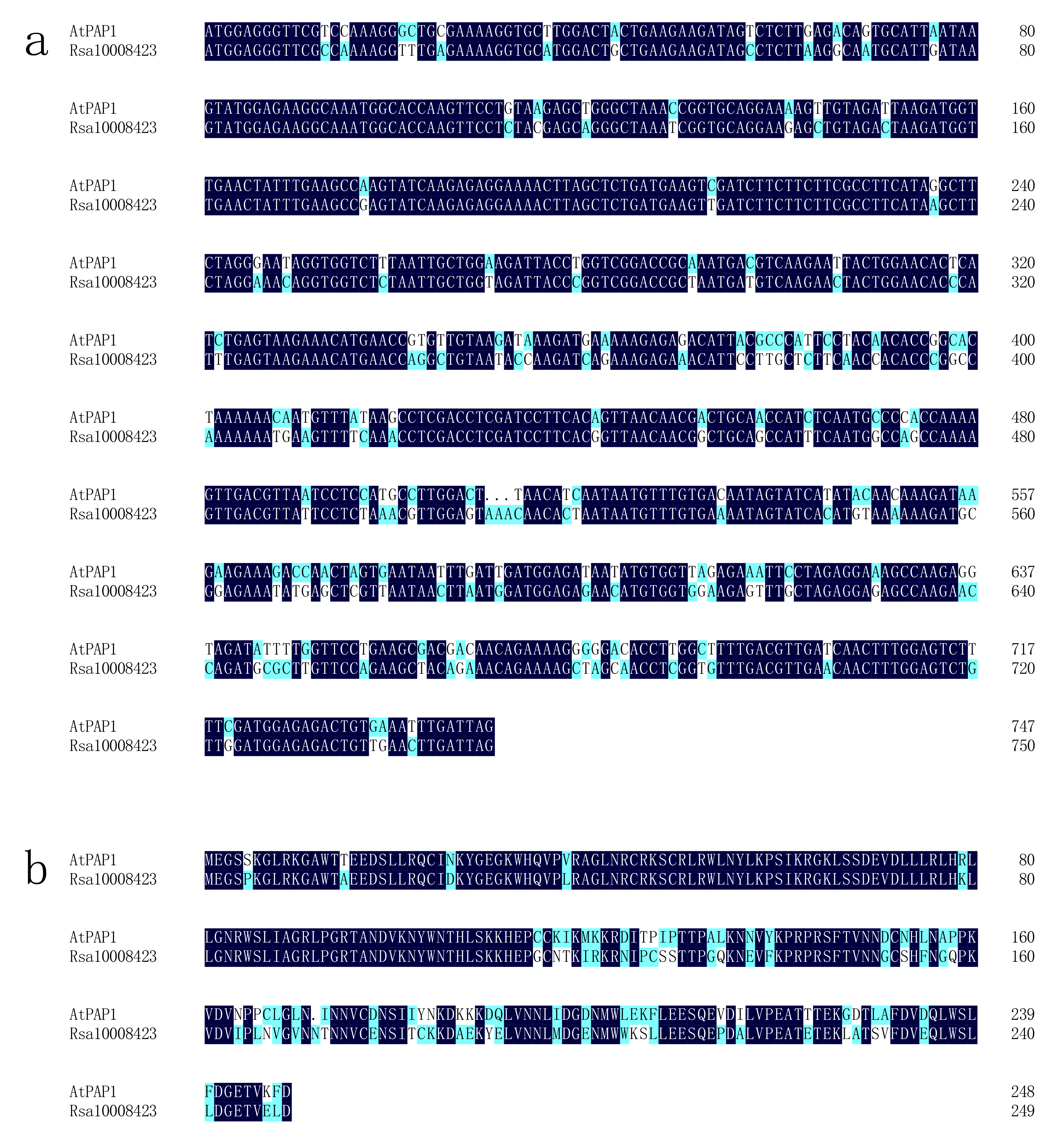

Supplement: Supplementary Figure 1 — Alignment of radish Rsa10008423 with the Arabidopsis PAP1 (MYB75, AT1G56650) gene coding sequence (a) and their deduced amino acid sequences (b). [file Image_1.jpeg]

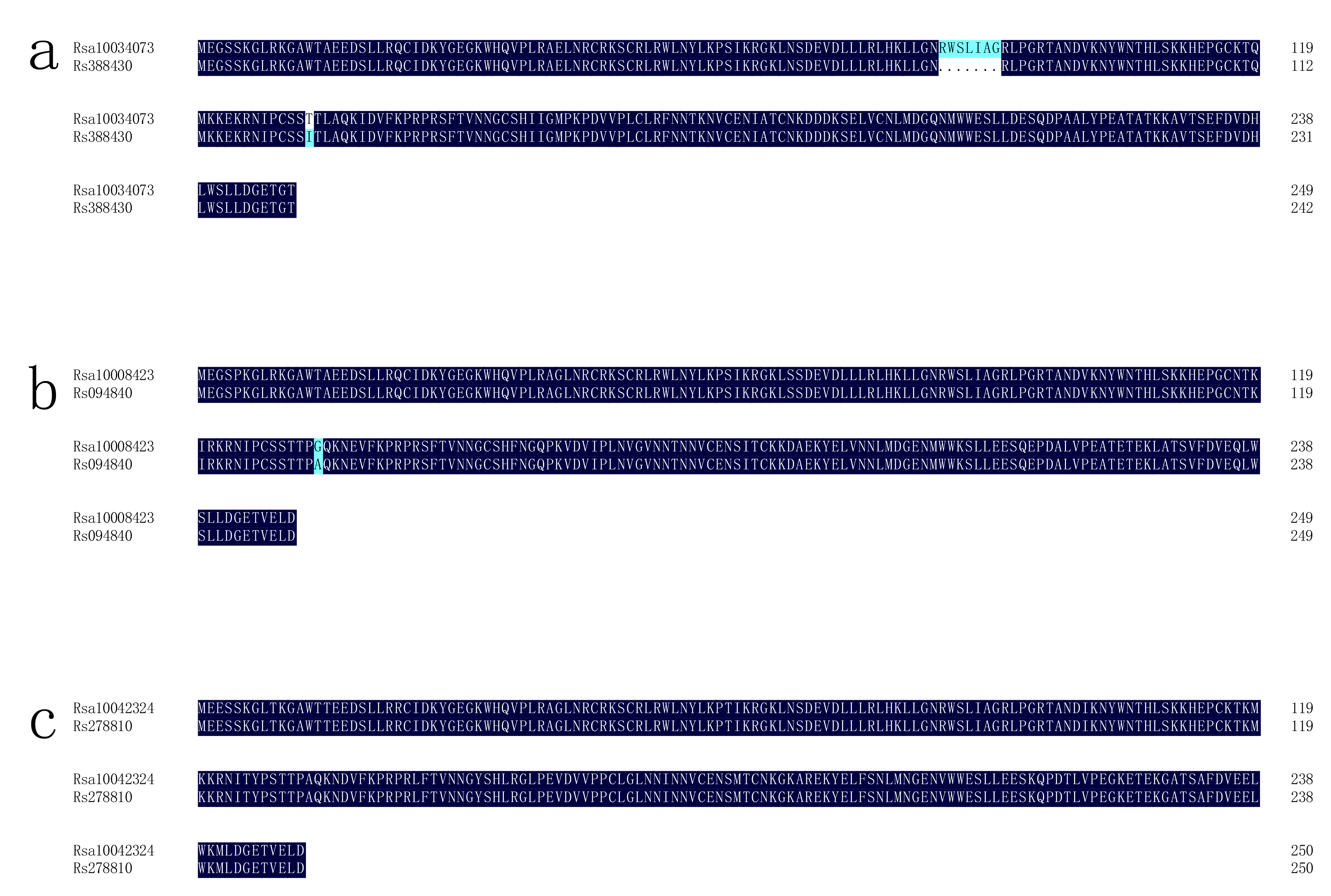

Supplement: Supplementary Figure 2 — Alignment of radish Rsa10034073 and Rs388430 (a), Rsa10008423 and Rs094840 (b), and Rsa10042324 and Rs278810 (c) based on their deduced amino acid sequences. [file Image_2.jpeg]
